# Molecular Mechanisms of Lipid Metabolism Disorders in Infectious Exacerbations of Chronic Obstructive Pulmonary Disease

**DOI:** 10.3390/ijms22147634

**Published:** 2021-07-17

**Authors:** Stanislav Kotlyarov, Anna Kotlyarova

**Affiliations:** 1 Department of Nursing, Ryazan State Medical University, 390026 Ryazan, Russia; 2 Department of Pharmacology and Pharmacy, Ryazan State Medical University, 390026 Ryazan, Russia; kaa.rz@yandex.ru

**Keywords:** COPD, exacerbations, immune response, inflammation, lipid metabolism, phagocytosis, lipid rafts

## Abstract

Exacerbations largely determine the character of the progression and prognosis of chronic obstructive pulmonary disease (COPD). Exacerbations are connected with changes in the microbiological landscape in the bronchi due to a violation of their immune homeostasis. Many metabolic and immune processes involved in COPD progression are associated with bacterial colonization of the bronchi. The objective of this review is the analysis of the molecular mechanisms of lipid metabolism and immune response disorders in the lungs in COPD exacerbations. The complex role of lipid metabolism disorders in the pathogenesis of some infections is only beginning to be understood, however, there are already fewer and fewer doubts even now about its significance both in the pathogenesis of infectious exacerbations of COPD and in general in the progression of the disease. It is shown that the lipid rafts of the plasma membranes of cells are involved in many processes related to the detection of pathogens, signal transduction, the penetration of pathogens into the cell. Smoking disrupts the normally proceeded processes of lipid metabolism in the lungs, which is a part of the COPD pathogenesis.

## 1. Introduction

Chronic obstructive pulmonary disease (COPD) is a chronic inflammatory disease, the prevalence and social significance of which is of increasing concern to clinicians and researchers [1,2,3,4]. It is shown that COPD is among the leading causes of morbidity and mortality worldwide [5,6]. As forecasts predict, the medical and economic burden of the disease will only grow.

Despite the available advances in COPD research, the keys to understanding all the pathophysiological mechanisms underlying both the disease itself and its comorbid interactions are still unknown. It is also not fully understood how one major etiological factor—cigarette smoking—can initiate the development of differences in clinical characteristics variants of COPD course.

Exacerbations are considered an important part of the clinical heterogeneity of COPD, they can modulate the nature of the course and mediate the relationship with comorbid diseases [7]. Exacerbations are events in the natural course of COPD, which are characterized by an increase in the severity of chronic respiratory symptoms (shortness of breath, cough, and sputum production) that go beyond their daily variability [8,9]. Exacerbations are associated with a deterioration in the quality of life and prognosis [10]. Taking into account the influence of exacerbations on the character of the course of the disease, some authors suggest that frequent exacerbations should be considered as a separate phenotype of COPD [11,12,13].

The mechanisms whose disorders are associated with exacerbations of COPD are of great clinical and research interest [14]. Exacerbations can be caused by many different factors, including viruses, bacteria, and aeropollutants [9,15,16]. It is shown that bacterial colonization of the bronchi makes a significant contribution to the progression of COPD [17].

The data accumulated in recent years leave no doubts that lipid metabolism disorders are widely involved in the pathogenesis of COPD [18,19,20]. These disorders occur at different levels and participate in both the development of inflammation and the formation of pulmonary and extrapulmonary clinical heterogeneity of the disease [21]. It is shown that lipid metabolism is connected with the development of COPD phenotypes, such as emphysema [22]. Interestingly, dyslipidemia causes multidirectional effects on innate immunity in the lungs and in the body in general, which is associated with the unique lipid biology of the lungs [23].

## 2. Lipid Metabolism and Its Disorders in COPD

Lipids play an important role in providing lung function. They perform not only a structural or energy function but also act as participants in many processes [24,25]. The lipid metabolism in the lungs is extremely complex, characterized by the participation of many types of lipids, and has specific features characteristic of different cell types. Some cells may even have their own characteristics of lipid metabolism associated with their differentiation and functional activity [26,27]. This complexly maintained lipid homeostasis of the lungs participates in providing an immune response and regulating inflammation.

The role of lipids in the immunometabolic reprogramming of macrophages is well known [28,29]. It is shown that the inflammatory activation of macrophages is largely related to the peculiarities of cellular metabolic processes and has a complex, not fully studied regulation [28,30]. Proinflammatory activated M1 macrophages are characterized by enhanced glycolysis and an increase in the synthesis of fatty acids, which are used as precursors for the synthesis of inflammatory mediators. In addition, they are characterized by a disturbed tricarboxylic acid (TCA) cycle and oxidative phosphorylation (OXPHOS). At the same time, anti-inflammatory M2 macrophages have intact TCA, OXPHOS, and enhanced fatty acid oxidation (FAO) [29,31,32,33,34].

The relative stability of the continuous processes of lipid metabolism in the cells of the respiratory tract is disrupted during smoking [19,20]. Circulating mononuclear cells in healthy smokers and patients with COPD demonstrate a reduced ability to utilize glucose. However, in patients with COPD, there is also a violation of the oxidation of fatty acids. In healthy smokers, the rate of fatty acid metabolism in mononuclear cells correlated positively with the parameters of lung function [32].

Tobacco smoke is a complex, not fully studied mixture of several thousand different chemicals, including many free radicals, which can have a significant effect on the lipids of the lungs [35]. Exposure to cigarette smoke causes lipid peroxidation in the bronchial epithelium and also leads to the redistribution of various lipid fractions [36].

Smoking leads to cytoplasmic accumulation of lipids [37] and also causes a decrease in the fluidity of the plasma membrane of alveolar macrophages, which is associated with a change in its lipid composition [38]. Exposure to cigarette smoke affects the metabolism of fatty acids in the cells of the respiratory tract, leading to a violation of the ratio of saturated and unsaturated fatty acids in the composition of phospholipids of the plasma membranes of cells [39].

The metabolism of fatty acids is disturbed both in a stable state and during exacerbations of COPD [40]. Moreover, it concerns both free fatty acids and fatty acids in the composition of phospholipids. With a stable course of COPD significantly lower levels of free alpha-linolenic acid, linoleic acid, and eicosapentaenoic acid are found in sputum compared to the control group [39,40]. It is shown that free polyunsaturated fatty acids (PUFA) have an antimicrobial effect, which is associated with a direct effect on the membranes of bacterial cells, as well as due to the formation of their bioactive metabolites that affect phagocytosis [41]. Interestingly, during COPD exacerbation a slight increase in the levels of PUFA (free arachidonic acid and docosapentaenoic acid) compared to stable COPD was being observed [39]. It should be noted that in contrast to ω-3 PUFA, ω-6 PUFA, such as arachidonic acid, increase inflammation in COPD [42,43].

The indicated changes in the lipid composition are a part of many processes connected with the fluidity of the plasma membrane, the organization of lipid rafts, the assembly, and functioning of receptor signaling pathways that provide cross-links of lipid metabolism, disturbed during smoking and COPD with microbial colonization of the bronchi and infectious exacerbations of the disease.

### 2.1. The Importance of Lipid Rafts in the Pathogenesis of COPD

A growing amount of evidence reinforces the understanding of the significance of plasma membrane lipid rafts in the interaction of pathogens with lung cells [44,45]. Lipid rafts are special structures of the plasma membrane of cells that are enriched with cholesterol and sphingolipids and act as dynamic platforms that recruit a number of signaling and transport proteins and provide many important cellular functions [46]. It should be noted that since the moment of its description [47], the concept of lipid rafts has been the subject of active discussions, including discussion of not only the structure and function of rafts but also of the very fact of their existence.

Cholesterol, the predominant component of lipid rafts, makes a significant contribution to their structural organization [48]. It is assumed that having a polycyclic sterane ring, cholesterol provides a more dense packing of lipids in rafts, which increases their viscosity compared to non-raft domains. Membrane proteins reside temporarily in lipid rafts and their function depends on localization in lipid rafts, including due to specific lipid–protein interactions [49,50]. Structural changes in lipid rafts and their disorganization caused by changes in the content of individual lipid components lead to changes in the function of the proteins associated with them. Saturated and polyunsaturated fatty acids have different effects on the structure and function of lipid microdomains [51,52].

Currently, two types of plasma membrane lipid domains are well characterized: flat lipid rafts enriched with glycosylphosphatidylinositol (GPI) and related to it proteins, and so-called caveols, which are invaginated into the cell microdomains, stabilized by structural proteins caveolins [53], among which the main framework protein is caveolin-1, which forms oligomeric structures and is present in many cell types, including epithelial cells of the respiratory tract [54]. It is assumed that caveols are more stable than flat lipid rafts. These lipid microdomains provide the implementation of many biological processes, including signal transduction, cellular metabolism, cell growth, and proliferation, apoptosis, and they also take part in the development of pulmonary inflammation [55,56,57,58,59].

It is assumed that the average size of lipid rafts varies from 10 to 200 nm [60], while the diameter of the caveol opening on the surface of the plasma membrane is 60–80 nm [61,62]. It is believed that caveols can have different forms, including flat, tubular or vesicular [63]. Despite a certain similarity in lipid composition, there is no convincing evidence of the possibility of mutual transition between caveols and flat lipid rafts [64].

The significance of caveolin-1 is emphasized by the fact that knockout of the Cav1 gene leads to the loss of caveols by cells [65] and the development of lung lesions characterized by thickening of the alveolar septa due to endothelial cell hyperproliferation and fibrosis [65]. Due to its ability to regulate NO production via endothelial NO synthase (eNOS), caveolin-1 participates in the activation of nuclear factor kappa-light-chain-enhancer of activated B cells (NF-kB) and the inflammatory response of the lungs to the lipopolysaccharides of the cell wall of gram-negative bacteria (LPS) [66].

It is assumed that cholesterol interacts directly with caveolin-1 thanks to the cholesterol recognition/interaction amino acid consensus domain (CRAC) [67]. Cholesterol depletion reduces the number of caveols, while excessive cholesterol saturation, on the contrary, leads to a decrease in the fluidity of plasma membranes and an increase in the number of caveols [68].

Cellular transport and export of lipids are subjects of close study, taking into account their significance for lung diseases [69]. This significance is well illustrated by the fact that the lungs are an organ with a high level of expression of the protein ATP binding cassette transporter A1 (ABCA1 transporter). ABCA1 is considered a key participant in the process of reverse cholesterol transport and provides the formation of HDL by removing excess cholesterol from macrophages, in a process called reverse cholesterol transport [70]. Smoking reduces the expression and functional activity of ABCA1, which leads to intracellular accumulation of cholesterol and inflammatory activation of macrophages [71,72].

Another fact that could demonstrate the important role of lipids in lung function is the data on the participation of ABCG1 in the inflammatory response. ABCG1, like ABCA1, is involved in lipid transport processes, including the formation of HDL. The transporter is highly expressed in alveolar macrophages and type 2 pneumocytes and carries out cholesterol export, reducing its intracellular accumulation. Experimental data showed that mice with a knockout of the Abcg1 gene have an enhanced inflammatory response to gram-negative bacteria in the lungs, characterized by the participation of neutrophils, as well as increased cytokine production, compared to wild-type individuals. Although the Abcg1 −/− mice showed an increase in bacterial clearance in the lungs and a decrease in extrapulmonary bacterial spread, this effect was not prognostically positive, since excessive inflammation caused the development of structural and functional changes in the lungs and led to a higher mortality rate among Abcg1 −/− mice [73].

The decrease in the expression of ABCA1 and ABCG1 in the lungs of COPD patients is well known from the results of in vitro and in vivo studies [72,74], which emphasizes the importance of lipid metabolism disorders in the pathogenesis of the disease.

The interest in ABC transporters is enhanced by the data on their key role in ensuring the asymmetry of plasma membrane lipids [75]. It is shown that the level of expression and functional activity of ABCA1 determines the distribution of phospholipids between the plasma membrane sheets [76,77,78,79] since the transporter can fulfill their transfer both from the inner to the outer sheet, and participate in their horizontal movement. ABCA1-mediated changes in the distribution of membrane lipids determine the physicochemical properties of the plasma membrane and are important for the spatial organization of membrane proteins and, accordingly, their functional activity.

An increase in expression and functional activity of ABCA1 leads to a significant redistribution of cholesterol and sphingomyelin from rafts to the remaining part of the plasma membrane, while caveolin is also redistributed from caveols to the general region of the plasma membrane [80]. Thus, ABCA1 contributes to the destabilization of lipid microdomains [80,81,82]. This information can be confirmed by experimental data, in which mice with ABCA1 deficiency showed increased lipid rafts and inflammatory cell activation, accompanied by increased TNF-alpha production, against the background of reduced lipid export from macrophages [82].

These data give a possibility to assess the role of ABC transporters and the violation of their function in smoking and COPD. Interestingly, LPS suppresses ABC-mediated reverse cholesterol transport in human macrophages [83], promoting the accumulation of cellular cholesterol and enhancing the inflammatory activation of macrophages.

Thus, lipid rafts ensure the assembly and functioning of many signaling pathways, including those that provide detection of extracellular pathogens, and the modulation of the lipid phase of the plasma membrane affects the organization and function of membrane proteins.

### 2.2. Participation of Lipids in the Regulation of TLR Signaling Pathways

It is shown that the immune surveillance and detection of a large number of pathogens in the lungs are carried out by representatives of the family of Toll-like receptor (TLR) macrophages. TLRs are expressed by many cell types, including epithelial cells, endothelial cells, monocytes, macrophages, dendritic cells, T- and B-lymphocytes. The most studied Toll-like receptor, TLR4, is localized both in the plasma membrane and in endosomes and recognizes LPS of gram-negative bacteria [84], which occupy a significant place in the structure of the causes of infectious exacerbations of COPD.

TLR4-mediated signaling is of great importance for COPD [85,86]. Interestingly, in addition to LPS, exogenous oxidants and components of tobacco smoke can activate TLR4 in the lungs [87,88,89].

Accumulating data are evidence of the important role of lipids in TLR4 function. It is shown that TLR4 is localized in the lipid rafts of the plasma membrane, the stability of which affects the function of the receptor [90]. Direct interaction of TLR4 with the cholesterol of lipid rafts is assumed. Thus, the regulation of TLR4 activity can be carried out through changes in the cholesterol content in the lipid rafts of the plasma membranes of macrophages [90].

Taking into account that the ABCA1 transporter is a key participant in cholesterol transport that affects the stability of lipid rafts, a decrease in its expression and functional activity during smoking leads to a decrease in the export of cholesterol from macrophages, which causes the initialization of inflammation by several mechanisms [91,92]. In its turn, TLR4 activation inhibits ABCA1 expression, which greatly reduces the outflow of cholesterol from macrophages [93,94].

Interesting data are that saturated fatty acids can also activate TLR4 [84,95,96]. Moreover, unlike saturated fatty acids, unsaturated ones do not have such an effect [96]. At the same time, several studies have shown the anti-inflammatory effect of ω-3 PUFA, which is realized through the inhibition of TLR4 signal transduction [97].

The biological function of this is not clear, but thanks to these and other data it is clear that TLR4 activation is modulated by endogenous lipids. Chemical modification of LPS with the replacement of saturated fatty acids with unsaturated ones eliminates the inflammatory activation of TLR [98].

Ceramide, whose elevated levels are detected in the lungs of smokers and COPD patients [99,100,101,102], also has the ability to activate TLR4 signal transfer in epithelial cells [103,104], due to its structural similarity to bacterial LPS [103,105]. Assembly and activation of the TLR4 receptor after binding of LPS to CD14 require the production of ceramide by acidic sphingomyelinase [106]. It is shown that LPS and palmitic acid contribute to the increase of ceramide production by increasing the hydrolysis of sphingomyelin by stimulating the activity of acid sphingomyelinase [107,108,109].

Ceramide is a participant in structural apoptosis of the alveolar epithelium and endothelial cells, as well as violation of efferocytosis by alveolar macrophages in the emphysematous lung [101,110]. In this regard, it is believed that these representatives of sphingolipids are involved in the development of emphysema [101], a frequent phenotype of COPD.

The presented studies confirm strongly the opinion about the important role of the lipid architecture of plasma membranes in the signaling function of TLR4 and its possible violations in the modification of this architecture.

Taking into account these and other facts, it is interesting to know how disorders of lipid metabolism in COPD patients are associated with infectious exacerbations and disease progression.

## 3. Bacterial Infection in the Pathogenesis of Stable Course and Exacerbations of COPD

The lungs are constantly in contact with a large number of microorganisms and aeropollutants when breathing. Taking into account the large surface area of the lungs and the volumes of air that a person inhales daily, the airways require a reliable functioning of the immune system. Interestingly, the bronchial tree of healthy people, which was previously considered sterile, actually contains a diverse microbial community [111,112,113]. Representatives of the genera *Pseudomonas*, *Streptococcus*, *Prevotella*, *Fusobacterium*, *Haemophilus*, *Veillonella*, and *Porphyromonas* are most often found in healthy airways [113,114,115,116,117,118,119]. It is assumed that in healthy people the lung microbiota is not a permanent community, but is represented by temporarily present microorganisms, the source of which is the upper respiratory tract [120,121,122,123]. It is known that even healthy people are characterized by microaspiration of the contents of the upper respiratory tract [124], whereas in COPD it and the damaging effect of smoking suppress additionally mucociliary clearance of the bronchi [125,126,127,128], which contributes to an increase in the population of the microorganisms into the lungs [120,129,130,131]. It should be noted that despite the fact that smoking disrupts the human immune response, there are no data on the direct effect of smoking on the microbiome of the lower respiratory tract [127,132,133]. Changes in the air flow in the bronchi, characteristic of COPD should also be related to the factors affecting the dynamics and character of the migration of microorganisms into the respiratory tract [124].

It is believed that representatives of the normal respiratory microbiome are metabolically active and participate in providing immune homeostasis in the respiratory tract [123,127]. By regulating the function of alveolar macrophages, the microbiota can enhance resistance to a variety of bacterial and viral pathogens [134]. Several studies have demonstrated changes in the structure of the microbiota in the lower respiratory tract during the transition of COPD from a stable state to an exacerbation, which may reflect the dynamism of the ongoing processes associated with the persistence of microflora [113,135,136,137,138,139,140,141,142].

Bacterial infections are an important cause of COPD exacerbations [113,143,144,145,146,147,148]. During exacerbation *Haemophilus influenzae*, *Moraxella catarrhalis*, *Pseudomonas aeruginosa*, *Klebsiella pneumoniae*, *Streptococcus pneumoniae*, *Staphylococcus aureus* are most often detected [116,117,119,135,149,150,151,152,153,154,155]. In a severe course of exacerbations in patients with severe stages of the disease, the structure of the microflora may change with the predominance of *Pseudomonas* spp. [116,141,143,153,156,157].

Bacteria can use different strategies for using lung lipids when colonizing the lungs [158,159]. Currently, extensive data on the involvement of host lipids in the metabolism of bacterial pathogens have been obtained. An important source of free phospholipids and sphingolipids in the human host organism for bacteria is the lung surfactant [160]. For example, *P. aeruginosa* uses phosphatidylcholine for growth [161,162,163].

In addition, with various tools associated with the assimilation of host lipids, their structural or stereochemical modification, bacteria have developed many tactics for using lipids to increase their virulence [159].

## 4. Cross-Links of Lipid Metabolism and Bacterial Infection in the Lungs

Recent studies demonstrate convincingly the ability of some viruses and bacteria to use the endocytic properties of lipid microdomains to enter cells [164,165,166,167,168]. It is also shown that cigarette smoke alters the function of several important endocytic pathways in the epithelial cells of the respiratory tract, increasing caveolin-mediated endocytosis, which may contribute to lung infection in smokers [169].

*P. aeruginosa*, an important participant in severe exacerbations of COPD, has the ability to enter epithelial cells through caveolin-rich lipid microdomains of the plasma membrane [57,170,171,172].

Several studies have shown that acidic sphingomyelinase and ceramide play an important role in the infection of cells with *P. aeruginosa* [173]. It was found that *P. aeruginosa* can initiate the activation of acidic sphingomyelinase, which hydrolyzes sphingomyelin [174,175] to form ceramide.

Due to their biophysical properties ceramide molecules self-associate and reorganize sphingolipid-rich rafts through hydrophobic interactions, creating larger microdomains with unique biophysical properties that are necessary for the transmission of diverse signals and clustering of several molecules, such as NADPH-oxidase, CD95, and cystic fibrosis transmembrane conduction regulator (CFTR) [171,176,177,178]. Interestingly, these ceramide-rich platforms are used by *P. aeruginosa* for internalization into the cell [171,177]. Genetic deficiency of acidic sphingomyelinase increases the susceptibility of mice to *P. aeruginosa*-induced pulmonary inflammation, generalization of infection, and death [171].

Activation of mitochondrial acidic sphingomyelinase is another tool for regulating *P. aeruginosa* inflammation, since the formation of mitochondrial ceramide increases the permeability and release of cytochrome C from mitochondria and causes neutrophil death [179,180,181]. By regulating the activity of acidic sphingomyelinase, *P. aeruginosa* not only participates in the reorganization of plasma membranes with the formation of platforms enriched with ceramides but also can influence other immune processes, taking into account the fact that ceramide and sphingosine take part in the protection of the lungs from bacterial pathogens [21].

The structural component of caveols, the protein caveolin-1, plays an important role in the immune defense of the lungs. It is shown that it is involved in the regulation of various signaling pathways and inflammatory responses associated with infection with *K. pneumoniae* [182] and *P. aeruginosa* [183,184]. Mice with a knockout of the Cav1 gene have an increased sensitivity to *P. aeruginosa*, which correlates with a reduced ability of neutrophils with caveolin-1 deficiency to phagocytize *P. aeruginosa* [185]. The significance of caveols is well demonstrated by a study that showed that mice with a knockout of the Cav1 gene infected with *P. aeruginosa* had increased mortality due to severe lung damage and generalization of infection compared to wild-type mice. In addition, these mice showed increased production of inflammatory cytokines (IL-6, TNF-α, and IL-1β) associated with the activation of the Cav-1/STAT3/NF-kB axis, as well as the reduced phagocytic capacity of macrophages and a marked increase in lipid peroxidation in the lungs [183].

It is known that ABCA1 co-localizes and interacts with caveolin-1, which initiates the oligomerization of caveolin-1, which is necessary for its intracellular transport from the Golgi compartment to the plasma membrane [186]. *P. aeruginosa* increases the expression of ABCA1, thereby being able to influence the transport of lipids [187]. This ability to influence a key lipid transport protein appears to be extremely important for the pathogenesis of COPD.

The Gram-negative bacterium *K. pneumoniae* can also enter lung epithelial cells through lipid rafts [182,188]. Cholesterol plays an important role in this interaction, and the reorganization of lipid rafts participates in protecting the host from *K. pneumoniae* infection [182,189]. The destruction of lipid rafts by methyl-β-cyclodextrin inhibits the internalization of *K. pneumoniae* into lung cells [182]. It should be noted that the biological function of internalization of *K. pneumoniae* by respiratory epithelial cells is a subject of discussion since on the one hand, it can be a useful mechanism for the pathogen, as it promotes bacterial replication and evasion of occupational phagocytes, while on the other hand, the respiratory epithelium is a participant in the antimicrobial defense of the lungs [190,191,192,193,194,195].

Experimental data show that cholesterol affects the expression of *K. pneumoniae* virulence factors, the key of which is the capsule polysaccharide, which provides resistance to phagocytosis [196,197]. It was found that cholesterol interferes with the formation of capsule polysaccharides, which increases macrophage-mediated phagocytosis but does not affect the adhesion of the bacterium to epithelial cells [188]. Depletion of cholesterol from cell membranes, on the contrary, stimulates the production of capsule polysaccharide, reducing phagocytosis of *K. pneumoniae* by macrophages. It has been shown that cholesterol reduces the antiphagocytic properties of the *K. pneumoniae* capsule, promoting the absorption of bacterium by macrophages [188].

In addition to the capsule polysaccharide, cholesterol is also able to suppress the expression of genes encoding LPS and *K. pneumoniae* outer membrane proteins. [188].

Fatty acids participate in the regulation of gene expression in some pathogens, and also affect their motility and survival. At the same time, polyunsaturated fatty acids have an inhibitory effect on *P. aeruginosa*. *P. aeruginosa* reacts to exogenous polyunsaturated fatty acids by changing the composition of phospholipids, membrane permeability, and phenotypes associated with virulence [198]. Linoleic and arachidonic acids although not synthesized by *P. aeruginosa*, but are included in the membrane phospholipids of the bacterium. Interestingly, arachidonic acid increases the resistance of the pathogen to certain antimicrobial drugs. It is assumed that the inclusion of arachidonic acid in the structure of the bacterial membrane may change its stereochemistry, with the help of enzymes such as cis/trans isomerase, which affects the permeability of the membrane and protects the bacterium from antimicrobial drugs [198,199,200]. Thus, the uptake and assimilation of arachidonic acid from the foci of infection may be beneficial for the colonization of *P. aeruginosa* [198] but may reduce the inflammatory response to infection due to impaired synthesis of eicosanoids [201,202,203].

*K. pneumoniae* is also able to assimilate fatty acids from the environment into its membrane phospholipids, which can affect its growth, virulence, and survival factors [204]. The length of the carbon chain and the degree of unsaturated fatty acid correlate with a decrease in bacterial growth [201]. At the same time, the inclusion of exogenous PUFAs leads to the destabilization of the bacterial membranes and increases their permeability for certain antimicrobial drugs. Interestingly, arachidonic acid promotes the colonization of *K. pneumoniae*, enhancing the formation of biofilms [201].

The lipid microdomains of plasma membranes are involved in the pathogenesis of other Gram-negative bacteria, such as *M. catarrhalis* [205]. Lipid rafts can be used not only for the penetration of bacterium into the cell but also for remote exposure to it through outer membrane-derived vesicles (OMV) in the absence of direct contact of bacteria with the host [205,206,207]. It is determined that the outer membrane-derived vesicles (OMV) secreted by *P. aeruginosa*, can deliver multiple virulence factors directly to the cytoplasm of epithelial cells of the respiratory tract through the fusion of OMV with the lipid rafts of the plasma membrane [206].

Thus, some bacteria can not only use the properties of plasma membranes for internalization into cells but also modulate their lipid structure and associated signaling pathways [181,208].

Modification of the fatty acid composition of membranes by bacteria is an important tool that affects the fluidity and permeability of bacterial membranes and, accordingly, the resistance to antimicrobial drugs [209,210,211].

## 5. Participation of Lipids in Phagocytosis Disorders in COPD

Phagocytosis is the most important mechanism that ensures the purification of tissues from foreign particles, microorganisms, and dead cells and is carried out by both professional and non-professional phagocytes [212,213]. The process of absorbing dead cells is called efferocytosis [214,215,216]. Macrophages are professional phagocytes and by their origin form two separate subpopulations in the lungs. Their number in the lungs in patients with COPD increases, which may be associated with increased recruitment of blood monocytes [217,218,219,220]. At the same time, both alveolar macrophages and macrophages differentiated from blood monocytes are characterized by defective efferocytosis and phagocytosis regarding a number of bacteria [221,222]. The phagocytic abilities of alveolar macrophages and macrophages derived from blood monocytes in COPD patients may differ, as was shown for *H. influenzae*, which provides an immunological basis for colonization of the respiratory tract by the bacterium in COPD [223,224] and may be one of the causes of exacerbation development [225]. In addition, the phagocytosis defect in COPD is mainly characteristic of bacterial pathogens, but not of inert microspheres [222].

Defective phagocytosis of bacteria is one of the factors leading to colonization of the lower respiratory tract and the development of infectious exacerbations of the disease [221]. Violation of phagocytosis by macrophages is associated with the frequency of exacerbations [225] and the severity of COPD, determined by indicators of pulmonary function [222,226]. Tobacco smoke is known to reduce the ability of alveolar macrophages to phagocytosis and efferocytosis [227,228]. Cells that died due to apoptosis and were not subjected to timely efferocytosis may be the cause of chronic inflammation [229]. Lipids oxidized under the influence of cigarette smoke make a significant contribution to local immune responses. It has been shown that exposure to cigarette smoke causes the production of antibodies against oxidized lipids in the lungs of mice, which may contribute to limiting the response to damaged lipids [230]. Interestingly, the phagocytic function of macrophages was dose-dependent suppressed by the presence of oxidized epithelial lipids [36].

Exposure to cigarette smoke leads to the accumulation of lipids in lung macrophages, with the formation of a foam cell phenotype [231]. It should be noted that the accumulation of cholesterol in macrophages may be facilitated by a decrease in the expression and functional activity of the ABCA1 transporter during smoking. The function of ABCA1 in phagocytosis is to remove excess cholesterol that is formed during uptake, for example, of apoptotic cells. At the same time, cholesterol-loaded macrophages are less effective phagocytes, which corresponds to data on a decrease in the phagocytic activity of ABCA1 deficient macrophages. In addition, ABCA1 participates in the “find-me” and “eat-me” signals, which are necessary for phagocytosis and efferocytosis [232].

Thus, phagocytosis is closely related to lipid metabolism. Active phagocytosis leads to an increase in the rate of lipid metabolism, which may reflect the general metabolic stimulation accompanying this process [233]. The increased synthesis of phospholipids during phagocytosis [234] is associated with the need for their use for the formation of membranes of phagocytic vesicles [235].

It is shown that the phagocytic activity of macrophages is determined by the composition of the plasma membranes of cells. At the same time, the ratio of saturated and unsaturated fatty acids in phospholipids is important [236]. Experiments with the cultivation of macrophages in the presence of various fatty acids have shown that they are well incorporated into the composition of plasma membranes, affecting phagocytic activity, while its greatest increase is observed in the presence of cis unsaturated fatty acids [237].

Some bacteria have developed certain strategies that allow them to avoid death during phagocytosis [238]. There is strong evidence that *K. pneumoniae* survives phagocytosis by macrophages by regulating phagosome maturation. *K. pneumoniae* controls the maturation of phagosomes by regulating the PI3K-Akt-Rab14 axis, so that the bacterium does not die in macrophages, but is located in a special intracellular compartment that does not merge with lysosomes [189].

In addition, *K. pneumoniae* can disrupt efferocytosis, so that neutrophils infected with the bacterium are excreted through efferocytosis less efficiently than uninfected neutrophils [239]. For this purpose, *K. pneumoniae* can use several mechanisms, including reducing exposure to phosphatidylserine (PtdSer) by increasing flippase activity [239,240]. The localization of PtdSer on the outer sheet of the plasma membrane is known to be the «eat-me signal» for macrophage receptors that provide uptake of apoptotic cells [241]. In the plasma membranes of living cells flippases are responsible for the transport of PtdSer from the outer leaf to the inner leaf, whereas, in apoptosis, PtdSer moves in the opposite direction [242]. *K. pneumoniae* contributes to the shift of the cell death pathway from apoptosis to necroptosis of infected neutrophils [239,243]. Thus, disruption of apoptosis due to PtdSer translocation modulation and activation of necroptosis are independent mechanisms of disruption of neutrophil efferocytosis in *K. pneumoniae*. This allows the bacteria to enter the interstitium, avoiding uptake by macrophages [239,240].

Thus, in COPD there is a violation of various phagocytosis mechanisms that can use bacterial pathogens for colonization.

## 6. Participation of Lipids in the Resolution of Inflammation in COPD

The information accumulated in recent years allows us to re-evaluate the variety of functions of lipids in various phases of inflammation. More and more evidence suggests that lipids are involved not only in the initiation of inflammation, such as prostaglandins and leukotrienes formed from arachidonic acid, but are also mediators of the highly organized phase of inflammation resolution [244,245,246]. Recently identified new families of mediators, called “specialized pro-resolving mediators” (SPM) play a key role in the active resolution of inflammation [247,248,249,250]. This class of endogenously produced bioactive lipids includes lipoxins, resolvins, protectins, and maresins, which are formed by oxygenation of ω-3 and ω-6 PUFA [251,252]. Moreover, lipoxins are synthesized by a series of enzymatic reactions from arachidonic acid [253,254], resolvins of the E series from eicosapentaenoic acid, and resolvins of the D-series and protecins, as well as maresins, are formed from docosahexaenoic acid [255,256,257]. Thus, PUFAs, if necessary released from membrane phospholipids, are an important source of not only pro-inflammatory, but also anti-inflammatory mediators [256,257,258,259].

The function of SPM is being studied actively, but the data available to date allow us to emphasize their significant role in inflammation, which is provided by the regulation of many lower signaling pathways [244,247,259,260,261]. SPM affect the decrease in the secretion of pro-inflammatory cytokines, and on the contrary, they contribute to an increase in the number of anti-inflammatory cytokines, through switching macrophages to the M2 phenotype, and also increase phagocytosis, which is important, taking into account that tobacco smoke stimulates macrophages pro-inflammatory [259,261,262,263].

Taking into account the importance of SPM in the resolution of inflammation, and the violation of this process in COPD, the role of lipid mediators is of great clinical interest [262,264,265,266,267,268]. The participation of resolvin RvD1 in tissue regeneration in emphysema caused by smoking has been shown [269]. It has also been determined that resolvins inhibit the production of pro-inflammatory cytokines TNF-α and IL-6 by alveolar macrophages in COPD, weakening the inflammatory effects caused by cigarette smoke [270,271]. SPM also improves bacterial clearance by activating phagocytosis, such as RvD1, which enhances phagocytosis of *P. aeruginosa* by neutrophils and macrophages [271,272].

Lipid mediators demonstrate dysregulation of concentrations in various biological substrates of COPD patients, including exhaled air condensate, bronchoalveolar lavage fluid, and blood serum. It was found that the levels of anti-inflammatory lipoxin LXA4 decrease in sputum in COPD patients during exacerbation, while there is an increased ratio of pro-inflammatory leukotriene B (4) (LtB (4)) to LXA4 (LtB (4)/LXA (4)) [273].

Taking into account the role of SPM in providing bacterial clearance in acute infection, it is interesting to consider the relationship of SPM with chronic persistent infection, which is characteristic of COPD [272]. In this regard, it should be noted that there are currently known examples of the use of local SPM production by some pathogens as a strategy for evading host immunity and survival [261,262,274]. It is shown that *P. aeruginosa* participates in the biosynthesis of SPM by activating cytosolic phospholipase A2, thereby increasing the available pool of arachidonic acid and then metabolizing it with the help of functional 15-lipoxygenase (15-LOX) [274,275,276,277].

Thus, taking into account the biological role of lipid mediators and experimental data on the effectiveness of SPM in resolving inflammation, overcoming immunosuppressive effects caused by tobacco smoke [267], this group of lipid mediators is of great interest from the point of view of finding new effective therapeutic strategies and developing drugs for the treatment of COPD [270,271].

## 7. Conclusions

**Figure 1:**
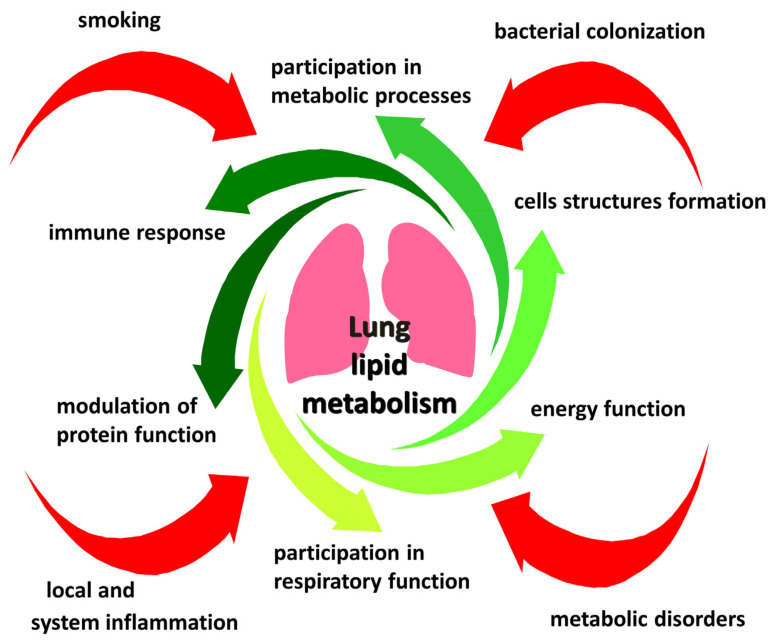
The role of pulmonary lipid metabolism.

The existing extensive evidence supports the significant role of lipid metabolism disorders in the progression of COPD (Figure 1, Table 1). Normal lipid metabolism is important for lung function, and its disturbances during smoking may participate in the pathogenesis of COPD [278]. The mechanisms of such disorders are multifaceted, associated with the processes of lipid peroxidation, defects in the synthesis and transport of lipids [279].

According to modern concepts, the bilayer of the plasma membrane is not homogeneous in its lipid composition but is represented by a mosaic of tightly packed lipid microdomains. Lipid microdomains perform many physiological functions, including the assembly and functioning of signaling pathways, such as TLR4, which provides LPS detection of Gram-negative bacteria.

Maintaining the membrane asymmetry of lipids is a complex, not fully studied mechanism in which many pathways of cellular lipid metabolism intersect, including their synthesis, absorption, export, and storage. The transport of lipids, including cholesterol, which is considered to be an important structural component of lipid microdomains, has complex regulation and can be disrupted during smoking. The substrate of such disorders may be a decrease in the expression and functional activity of the ABCA1 transporter. Modified by changes in the transport activity of ABCA1 the lipid environment in which TLR4 is embedded may underlie functional disorders caused by smoking.

The conducted analysis showed that lipid rafts are involved in the pathogenesis of COPD, as they provide many important cellular functions and participate in the immune response. Disruption of the structure and function of lipid rafts in smoking and COPD is part of the pathogenesis of COPD. These disorders participate in bacterial colonization of the bronchi, lead to an increase in inflammation.

A growing body of evidence leaves no doubt that some bacteria and viruses use the lipid microdomains of the plasma membrane for interaction, binding, and possible penetration into cells [164,165]. Some pathogens can affect the lipid metabolism in the host cells by realizing their virulence, as well as using the host lipids as a food source or assimilating them into their metabolic or structural processes [280]. For colonization of the lungs in COPD bacteria use many other strategies, including modification of the lipid composition of the membrane, which affects its permeability for antibacterial drugs, the formation of biofilms, and the exclusion of certain lipids from the host metabolic and immune processes [281].

It should be noted that not only the natural course of COPD determines all the features of the lipid metabolism of the lungs. These processes can be modulated under the influence of certain medications used to treat both the underlying disease and comorbid pathology. Information about the role of inhaled corticosteroids (ICSs), which can be used in COPD, is interesting [282,283]. It is believed that they are most effective in eosinophilic exacerbations of the disease, especially when combined with bronchial asthma (asthma-COPD overlap), but their use is ambiguous in infectious exacerbations of COPD when ICSs can increase the risk of pneumonia [283,284,285]. This may be due to a complex effect on various phases of inflammation [286]. Glucocorticoids affect the lipid metabolism of the lungs, due to their ability to inhibit the activity of phospholipase A2 [287], the enzyme responsible for the formation of arachidonic acid [288] and, accordingly, many inflammatory mediators [289,290], including the synthesis of prostaglandins, leukotrienes, and lipoxins [291]. Modulation of the synthesis of lipid mediators can change the immune response and the character of the course of infectious exacerbations in COPD.

Taking into account the significant role of lipid metabolism in lung function and disorders of this metabolism in COPD, it is logical to assume a positive role of taking statins, which have an anti-inflammatory pleiotropic effect [292,293,294,295]. However, studies conducted on this topic do not give a clear answer on the effectiveness of taking statins. They were unable to demonstrate a clear improvement in lung function and mortality from COPD [293,294,296,297], although they confirmed a decrease in the level of inflammation and a decrease in the negative impact of cardiovascular comorbidity [294,297,298]. The effect of statins on the frequency and severity of exacerbations is also a subject of discussion, although in some studies the use of this group of drugs has been associated with a reduced risk of hospitalizations associated with exacerbations [294,298,299,300,301,302].

The anti-inflammatory effect of statins includes various mechanisms, including the effect on the function of macrophages, direct antibacterial activity [303,304,305,306,307,308]. The effect of statins on the biophysical properties of plasma membranes and the function of membrane proteins is also shown, which is undoubtedly the subject of further research [309,310].

Thus, currently available data suggest that the leading role of lipid metabolism in the lungs is not only as a structural or energy substrate but also as a full-fledged participant in the immune defense of the lungs. The multifaceted role of lipids in the function of the immune defense of the lungs is only beginning to be understood. It can be assumed that lung lipids are at the intersection of many pathways of the innate immune response. Thus, the lipid metabolism in the lungs is a complex system, the keys to understanding all the mechanisms of which are still not available to clinicians and researchers.

## Figures and Tables

**Table 1:** Lipids disorders in COPD.

Lipids	Disorders That Occur during Smoking and COPD	Processes Affected by Lipid Metabolism Disorders	References	
Fatty acids	Disorders in the ratio of saturated and unsaturated fatty acids in the composition of membrane phospholipids;	activation and resolution of inflammation (lipid mediators of inflammation); activation of TLR4 signaling;	[20,28,29,30,32,33,34,35,38,39,40,41,42,43,52,95,96,108,234,235,236]	
	change in the amount of free fatty acids;	immuno-metabolism of macrophages (fatty acid oxidation)		
	fatty acid oxidation disorders			
Phospholipids	Disorders in the ratio of saturated and unsaturated fatty acids in the composition of membrane phospholipids	the fluidity and permeability of plasma membranes;	[19,24,38,39,40,51,52,234,235,236,237]	
		participation in ensuring the structure and function of the plasma membrane;		
		organization of lipid rafts;		
		regulation of the membrane protein function, including those associated with inflammation;		
		participation in phagocytosis		
Sphingolipids	Smokers and patients with COPD have elevated levels of ceramides in the lungs	increased structural apoptosis;	[19,20,21,24,35,99,100,101,102,103,104,105,106,107,109,110,176,177,178]	
		activation of TLR4 signaling;		
		organization of lipid rafts, reorganization of lipid rafts enriched with ceramide		
Cholesterol	Disorders of reverse transport of cholesterol from macrophages during smoking and COPD	participation in ensuring the structure and function of the plasma membrane;	[19,48,49,50,67,68,82,90,91,92,231]	
		organization of lipid rafts;		
		regulation of the function of membrane proteins, including those associated with inflammation;		
		phagocytosis disorders		

## Data Availability

Not applicable.
